# Influence of PARP1 on CRISPR/Cas9 induced double strand break repair in proliferating cells

**DOI:** 10.1016/j.csbj.2025.10.001

**Published:** 2025-10-02

**Authors:** Tobias Wimmer, Judith Schneider, Fatimah Alabudeeb, Franziska M. Türk, Lyubomyr Lytvynchuk, Knut Stieger

**Affiliations:** Department of Ophthalmology, Justus-Liebig-University Giessen, Germany

**Keywords:** *PARP1*, CRISPR/Cas9, DNA Repair, Cellular Reporter Assay

## Abstract

Genome editing with CRISPR/Cas9 depends on the induction of site-specific DNA double-strand breaks (DSBs), which are repaired by distinct pathways. Non-homologous end joining (NHEJ) frequently introduces insertions or deletions (indels), resulting in mutagenic repair, whereas microhomology-mediated end joining (MMEJ) produces larger deletions due to end resection. In the presence of a donor template, homologous recombination (HR) enables precise sequence changes but is generally inefficient in many cell types. Modulating the activity of key repair factors has therefore emerged as a promising strategy to bias DSB repair outcomes toward more predictable or precise edits. Here, we employed luminescent and fluorescent reporter assays to systematically quantify the impact of PARP1 modulation on repair pathway choice. We show that PARP1 downregulation increased both NHEJ and MMEJ repair without altering HR, while PARP1 overexpression reduced NHEJ and HR but left MMEJ activity unaffected. These results highlight PARP1 as a regulator of DSB repair balance and suggest that targeted modulation of PARP1 could improve the precision of CRISPR-based genome editing.

## Highlights

• PARP1 Modulates DNA Repair Pathway Choice.
• PARP1 Overexpression Suppresses Mutagenic Repair.
• PARP1 Depletion Enhances MMEJ but Also Promotes NHEJ.
• HDR Efficiency Remains Unaffected by PARP1 Downregulation.

## 1 Introduction

**Fig. 1:**
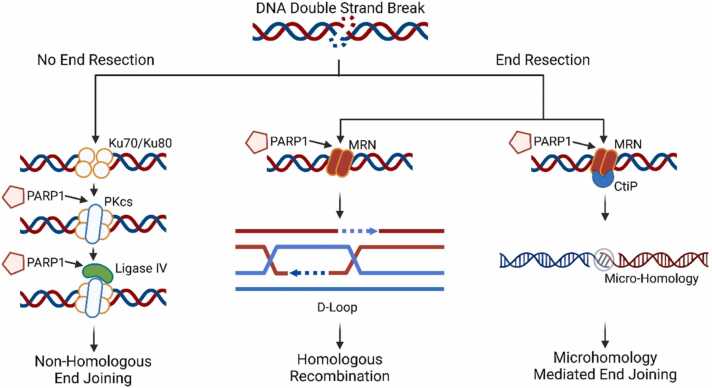
**PARP1 regulates DNA double-strand break repair.** At unresected DNA ends, Ku70/Ku80 directs c-NHEJ, while PARP1 modulates DNA-PKcs and Ligase IV. With end resection, PARP1 promotes MRN recruitment, enabling repair by HR or MMEJ through CtIP and short microhomologies. PARP1 thus interfaces with all major DSB repair pathways and influences pathway fidelity.

Therapeutic genome editing relies on the induction of DNA double-strand breaks (DSBs) by site-specific endonucleases, followed by their resolution through endogenous DNA repair pathways. Among these systems, CRISPR/Cas9 has become the most extensively investigated tool. DSBs are primarily processed by three competing mechanisms, homologous recombination (HR), canonical non-homologous end joining (c-NHEJ), and microhomology-mediated end joining (MMEJ or alternative NHEJ), whose activity is influenced by the cell cycle phase and chromatin context. [1] Poly(ADP-ribose) polymerases (PARPs) catalyze poly(ADP-ribosyl)ation (PARylation) of themselves and other proteins using NAD⁺ as substrate. [2] PARylation is a critical post-translational modification that regulates DNA repair pathway choice. Among the PARP family, PARP1, PARP2, and PARP3 are key mediators of both single-strand break (SSB) and double-strand break (DSB) repair. [3] At a DSB, PARP1 interacts with and modulates the structure of DNA-PKcs, thereby influencing intracellular NHEJ activity. PARP1 knockout studies in mice have revealed hyperactive NHEJ in bone marrow cells compared to wild-type cells, in which HR normally predominates. [4], [5] PARP1 also contributes to MMEJ by promoting complexation with Ligase III and XRCC1 at DSB sites. [6] Although PARP1 is not essential for HR, it plays a role in stabilizing the replication fork and recruiting HR-associated repair proteins such as RAD51 and MRE11. [7] Consequently, PARP1 is implicated in all three major DSB repair pathways (Fig. 1).

Luminescent and fluorescent reporter systems, though not widely applied in DNA repair research, offer valuable insights into various aspects of genome editing. These systems typically rely on the correction or disruption of an endonuclease target sequence in an open reading frame. [8], [9] Several different variations of reporter assays to quantify DSB repair pathway choice, assess indel formation activity, or repair template integration efficiency have been described so far that rely on these principals. Simple reporter systems rely on the correct integration of a repair template into the coding sequence of a previously cleaved target site, this can be either a fluorophore for a qualitative assessment or luciferases, which are quantifiable. [10] Split-fluorescence assays that rely on the restoration of a disrupted fluorophore are used to indicate DSB repair, others like the traffic light reporter system can differentiate between NHEJ and HDR repair events. [11], [12] Bioluminescence Resonance Energy Transfer (BRET) based reporter systems are using an active substrate converting luciferase to excite an acceptor fluorophore, which subsequently emits light at higher wavelength again. The ratio of the luciferase- and the fluorophore-light output can be represented as the BRET ratio. Again, a restoration and a disruption of the acceptor’s coding frame is used to quantify the desired repair outcome. The BRET reporter is also capable to quantify both NHEJ and HDR mediated DSB repair by the addition of a repair template HDR, but it offers several additional advantages over fluorescent reporters. The light output of the luciferase can be used as an internal standard eliminating experimental fluctuations, thus reducing background noise. Furthermore, the possibility of repeated *in-vivo* measurements over several days with various experimental setups, along with the availability of *in-vivo* luciferase substrate formulations that do not require external excitation of a fluorophore, which can cause autofluorescence or cytotoxic photobleaching, enhances experimental flexibility and accuracy. [13], [14]

## 2 Material and Methods

### 2.1 Vector Generation

The BRET reporter plasmid was linearized using *AvrII* and *BsiWI* (NEB, Frankfurt, Germany). Cas9 target sequences containing single-stranded overhangs complementary to the restriction enzyme sites were designed and synthesized as oligonucleotides (Metabion, Planegg, Germany). After hybridization at 95°C for 10 min followed by incubation at room temperature for 30 min, these inserts were ligated into the linearized BRET reporter plasmid. Successful integration was confirmed by Sanger sequencing (Seqlab Microsynth, Göttingen, Germany).

Cas9 target corresponding Guide RNA (gRNA) sequences were cloned into the px459 plasmid (Addgene: #62988) under the control of a U6 promoter. The px459 plasmid also encodes the *staphylococcus pyogenes Cas9* (spCas9) nuclease. [15] Oligonucleotides were synthesized, hybridized as described above, and inserted into the plasmid via *BbsI* (NEB, Frankfurt, Germany) restriction sites. Correct gRNA sequence integration was verified by Sanger sequencing (Seqlab Microsynth, Göttingen, Germany).

### 2.2 Cell Culture

HEK293-T cells (ATCC: CRL-3216) were maintained in a humidified incubator at 37°C and 5 % CO₂ in Dulbecco’s Modified Eagle Medium (DMEM) (Anprotec, Bruckberg, Germany) supplemented with 10 % fetal bovine serum (FBS; PAN Biotech, Aidenbach, Germany), 4 mM L-glutamine (PAN Biotech), and 100 IU/ml penicillin and 0.1 mg/ml streptomycin (Anprotec, Bruckberg, Germany). Cells were sub-cultured at a 1:10 ratio using Accutase (Anprotec, Bruckberg, Germany) upon reaching confluence.

### 2.3 Transfections

A total of 200,000 cells per well were seeded in 6-well plates (Greiner, Frickenhausen, Germany) one day prior to transfection. After overnight incubation, the culture medium was replaced with 1.5 ml of fresh supplemented DMEM. Plasmid DNA was diluted in 100 µl sterile 150 mM NaCl, followed by the addition of 200 µl polyethylenimine (PEI; 1.0 mg/ml). The transfection mixture was vortexed and added dropwise to the cultured cells. After 4–6 h, the culture medium was replaced with fresh DMEM and further incubated in a humidified incubator at 37°C and 5 % CO₂.

### 2.4 Cell Lysis

Cells were washed once with 1x phosphate-buffered saline (PBS) and collected using a cell scraper in 100 µl of 1x PBS. Lysis was performed by two freeze-thaw cycles in liquid nitrogen**.** Samples were cleared by centrifugation at 17,000 x g for 5 min at 4°C**,** and the supernatant was either used immediately or stored at −20°C.

### 2.5 PARP1 (Human) Enzyme-Linked Immunosorbent Assay (ELISA)

Intracellular, human PARP1 levels were quantified using an ELISA kit (Biomatik, Heidelberg, Germany) according to the manufacturer’s instructions. Briefly, PARP1 knockout plasmids were transfected into HEK293-T cells, and after 48 h, cells were lysed via two freeze-thaw cycles in RIPA buffer containing protease inhibitors (1x Protease Inhibitor Cocktail; Sigma Aldrich, Darmstadt, Germany). The lysates were cleared by centrifugation at 1500 x g, and 100 µl of each sample was analyzed in triplicate at 450 nm. Data are represented as the percentage of PARP1 expression relative to untreated wild-type (WT) cells.

### 2.6 BRET reporter assay (frameshift rate)

The BRET (Bioluminescence Resonance Energy Transfer) reporter assay, based on NHEJ repair, was used to determine the frameshift rate (%) following Cas9-induced DSBs in the target sequence of the BRET reporter plasmid. [16] HEK293-T cells were transfected with 2.0 µg of the gRNA/Cas9-expressing px459 plasmid and 2.5 µg of its corresponding BRET target sequence vector. After 24 h incubation in a humidified incubator at 37°C and 5 % CO_2_, cells were washed once in 1x PBS, lysed, and processed as already described above. 10 µl of each lysate was plated in quadruplicate into white 96-well plates (COSTAR Lumiplates FlatWhite, Corning, Germany). After automated injection of 100 µl Coelenterazine 400a (1.0 µg/ml; Nanolight Inc., Pinetop, USA), luminescence and fluorescence was measured using transmission filters for blue (370–450 nm) and green (510–540 nm) wavelengths.

### 2.7 NHEJ assay

HEK293-T cells were transfected with the reference BRET reporter plasmid containing a characterized murine *CLN3* target sequence (5′-TGTGGGGCTTGCTCACCTCCAGG-3′) alongside with its corresponding gRNA/Cas9 plasmid. The reference BRET ratio from the *CLN3* reporter plasmid was used to calculate the indel formation rate change (%) after co-transfection with PARP1 knockout or overexpression plasmids.

### 2.8 HDR assay

The HDR assay followed the same protocol as the NHEJ assay, with the additional co-transfection of a linear pcr generated HDR template (0.5 µg) containing homologous sequences flanking a non-addressed target sequence. BRET reporters luminescence and fluorescence was measured as already described above in the BRET reporter assay section.

### 2.9 MMEJ assay

The MMEJ assay quantified the integration efficiency of an MMEJ repair template containing the open reading frame of *RLuc8* flanked by micro homologous sequences (MHSs) of varying lengths (10 bp, 15 bp, and 30 bp) into a stably integrated murine *RPGR ORF15* locus. HEK293-*mRPGR-ORF15* cells were seeded at 1 × 10⁵ cells per well in 6-well plates. Cells were transfected with two gRNA/Cas9 plasmids targeting the 5 ´ - and 3 ´ - Region of the ORF15 along with the MMEJ repair template. After 24 h, cells were lysed, and luminescence (photons/sec.) was recorded after automated injection of 100 µl coelenterazine 400a (1.0 µg/ml; Nanolight Inc., Pinetop, USA) with open transmission filters. [10]

### 2.10 Fluorescence microscopy

GFP2 fluorescence from BRET reporters was captured using a Keyence BZ-100 fluorescence microscope (Keyence, Neu-Isenburg, Germany) with an exposure time of < 0.5 s.

### 2.11 Polymerase Chain Reaction (PCR)

The HDR repair template was amplified via PCR using a BRET reporter plasmid with a non-addressed target sequence with primers (RLuc8 for: 5′-CTAGGCAGGCCCTTGTCGGAC TGCTGG-3′; GFP2 rev: 5′-GTACCCAGCAGTCCGAACAAGGGCTGC-3′) located in the *RLuc8* and *GFP2* coding sequence. The resulting 600 bp fragment was analyzed via agarose-gel-electrophoresis, further purified for mammalian transfection and used in the HDR assay experiments.

### 2.12 Statistical analysis

All data are presented as mean ± standard deviation (SD) from three independent experiments (n = 3) with quadruplicate measurements. Statistical significance was determined using t-tests or ANOVA, calculated with SigmaPlot (Systat, Erkrath, Germany). P-values *p < 0.05 were considered as statistically significant, and ***p < 0.001 was considered highly significant. BRET ratios were calculated as I_GFP2_/I_RLuc8_, with the RLuc8 background BRET ratio used for data normalization.

## 3 Results

### 3.1 Quantification of the on-target activity for the generated for PARP1 knockout gRNA/cas9 complexes

**Fig. 2:**
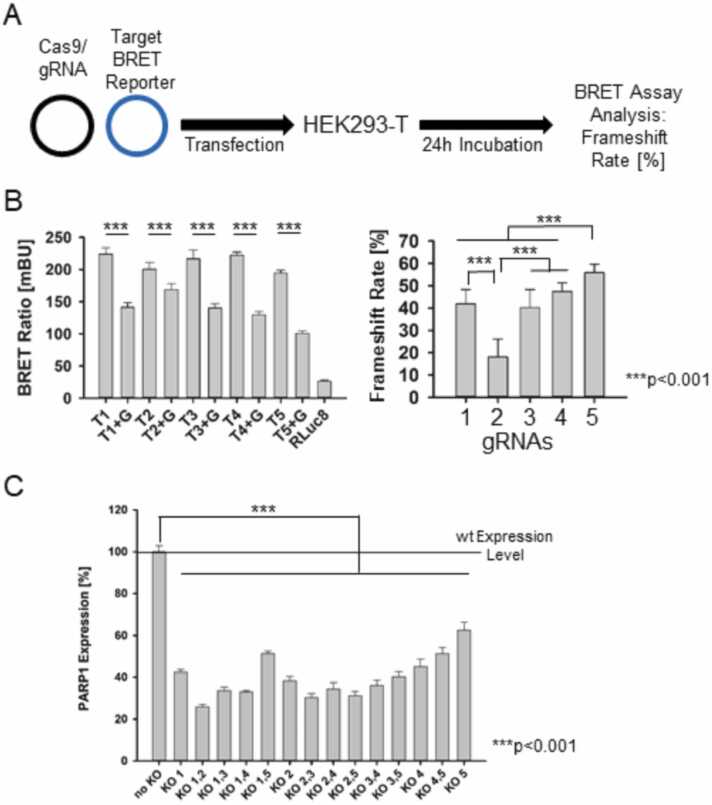
**Verification of PARP1 downregulation,** (A) Principle of the experimental setup using the BRET reporter assay. (B) BRET ratio of five different gRNA/Cas9 contructs on-target activity compared to the uncleaved BRET reporter carrying the gRNA/Cas9 target sequence and the transformed frameshift rate [%]. (***p < 0.001) (B) Downregulation of PARP1 expression after CRISPR/Cas9 PARP1 knockout plasmid transfection of HEK293-T cells compared to wild-type level, measured with a PARP1 specific ELSIA. (***p < 0.001).

Since PARP1 was the primary target of this study, we *in-silico* identified multiple gRNA/Cas9 target sequences within the human *PARP1* gene to induce a knockout by disrupting the reading frame. Gene knockout was achieved by the introduction of insertions, deletions, or mutations at the DSB site, thereby altering the original reading frame. The gRNA/Cas9 construct plasmids were co-transfected into HEK293-T cells and after a 24 h incubation and cell lysis the BRET assay was performed to characterize the on-target activity of five gRNA/Cas9 complexes. (Fig. 2A) We measured the reduction of the BRET ratio for each complex, compared to its non-treated control and calculated the resulting frameshift rates, which ranged from approximately 20–60 %. The lowest frameshift rate was observed for gRNA 2 (18.0 ± 8.0 %), whereas the remaining four constructs exhibited rates above 40 %, with gRNA 5 showing the highest rate at 55.9 ± 3.7 %, making gRNA 1, 3, 4, & 5 suitable for subsequent knockout PARP1 experiments (Fig. 2B). To confirm the knockout potential of the selected gRNA/Cas9 constructs, we employed a PARP1 ELISA following single or double transfections of the generated knockout plasmids into HEK293-T cells. The ELISA results demonstrated a highly significant (***p < 0.001) (compared to the non-treated control) reduction in PARP1 protein expression for all tested constructs and their combinations (Fig. 2C).

### 3.2 Influence of PARP1 Knockout and Overexpression on NHEJ Activity

**Fig. 3:**
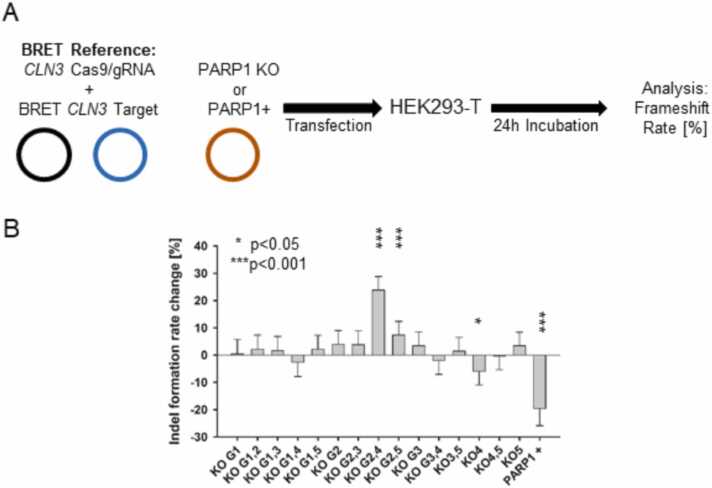
**Effect of PARP1 modulation on NHEJ activity.** (A) Schematic of the BRET-based NHEJ assay measuring indel formation after co-transfection of HEK293T cells with the BRET reporter *CLN3* reference and PARP1 knockdown or overexpression plasmids. (B) Indel formation rates [%] were significantly increased by PARP1 knockout and reduced by PARP1 overexpression (*p < 0.05; ***p < 0.001).

Following the confirmation of the on-target activity of the PARP1 gRNA/Cas9 knockout plasmids, we next examined the impact of PARP1 depletion and overabundance on NHEJ activity using the BRET reporter assay in HEK293-T cells. For reference we used a *CLN3*-BRET-Target with its corresponding gRNA/Cas9 complex with a known on –target activity and investigated the influence of PARP1 modulation on NHEJ DSB repair with the BRET assay after co-transfection in HEK293-T cells (Fig. 3A). The changes in the BRET reporter’s frameshift rate upon PARP1 modifications was calculated as the change rate [%] from the non-modified control and represented as the indel formation change rate. Among the different knockout conditions, the combination of KO plasmids 2 & 4 and KO plasmids 2 & 5 resulted in a highly significant (***p < 0.001) increase in NHEJ activity **(**23.8 ± 10.2 % and 7.4 ± 4.8 %, respectively). However, single knockouts did not significantly alter NHEJ activity, except for KO plasmid 4, which led to a slight reduction, although with low statistical significance (*p < 0.05)**.** In contrast, PARP1 overexpression led to a highly significant (***p < 0.001) reduction in NHEJ activity **(**19.7 ± 5.8 %**)**, demonstrating an opposing effect compared to the knockout conditions (Fig. 3B). This indicates hyperactive NHEJ with PARP1 downregulation, while PARP1 upregulation shows a decrease in NHEJ activity at the DSB site.

### 3.3 PARP1 modifications and MMEJ activity

**Fig. 4:**
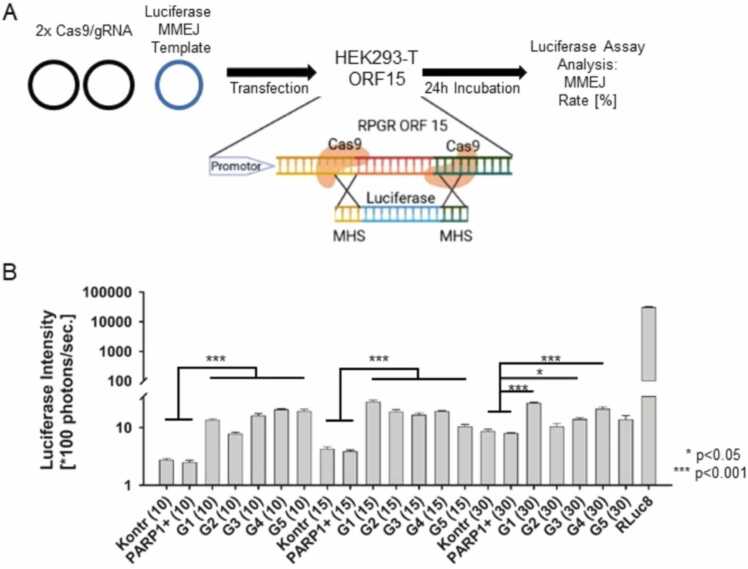
**Impact of PARP1 modulation on MMEJ-driven template integration** (A) Schematic of the MMEJ assay showing Cas9-mediated double cutting of the stably integrated *RPGR* ORF15 locus in HEK293 cells and co-integration of luciferase templates carrying microhomology sequences (MHS) of different lengths. (B) MMEJ integration frequency, measured as luciferase intensity, increased upon PARP1 downregulation and decreased upon PARP1 overexpression in an MHS length–dependent manner (*p < 0.05; ***p < 0.001).

To assess the influence of PARP1 expression on MMEJ, we utilized a previously described custom-made cellular MMEJ luciferase assay. In this experimental setup, HEK293-ORF15 cells with a stable genomic integration of the murine *RPGR* ORF15 locus were targeted at both the 5′- and 3′-ends with two gRNA/Cas9 contructs. To direct repair toward the MMEJ pathway, a luciferase reporters MMEJ repair template carrying microhomology sequences (MHS) of 10, 15, or 30 bp were co-transfected, and repair efficiency was subsequently evaluated by measuring luciferase activity after 24 h (Fig. 4A). The influence of PARP1 modifications on MMEJ template integration efficiency compared to the control was quantified via luciferase activity. While PARP1 overexpression did not significantly affect MMEJ integration efficiency at any of the MHS lengths tested, all PARP1 knockout conditions led to a highly significant increase (***p < 0.001) in MMEJ integration efficiency (Fig. 4B). An exception was observed for gRNA/Cas9 construct 3 in combination with the 30 bp MHS, which resulted in a less pronounced increase (*p < 0.05). Our data indicate here that PARP1 downregulation increases MMEJ repair template integration efficiency while PARP1 upregulation did not affect MMEJ repair pathway activity.

### 3.4 PARP1 expression and its effect on HDR activity

**Fig. 5:**
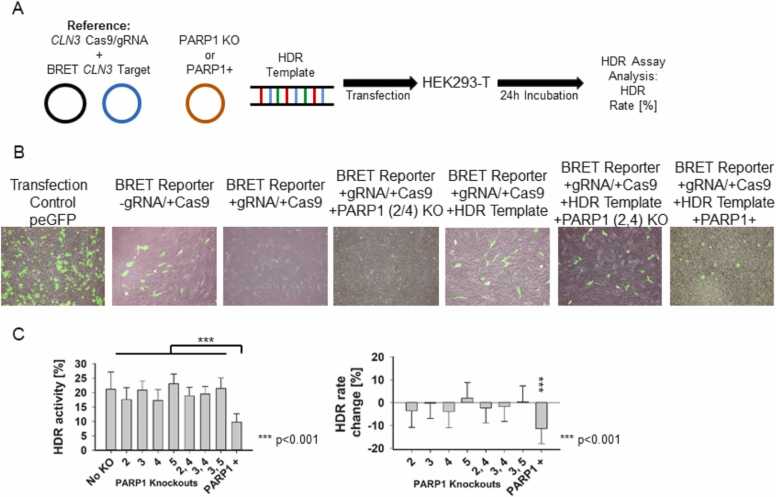
PARP1 modulation on HDR activity. (A) Schematic of the HDR assay in HEK293T cells co-transfected with the BRET reporter, an HDR template, and plasmids for PARP1 knockout or overexpression. (B) GFP2 fluorescence of BRET reporters under non-cleaved (−gRNA/Cas9), cleaved (+gRNA/Cas9), PARP1 downregulation (+gRNA/Cas9, +PARP1 KO), and HDR template conditions (+gRNA/Cas9, +HDR template), and the effect of PARP1 modulation in the presence of the HDR template. (C) Quantification of HDR activity and relative changes in HDR rate upon PARP1 downregulation or overexpression (***p < 0.001).

To determine the impact of PARP1 modulation on HDR**,** HEK293-T cells were co-transfected with our already used and described BRET-reporter *CLN3* reference system with a characterized gRNA/Cas9 activity pattern**.** PARP1 knockout or overexpression plasmids, and an additional HDR repair template containing 375 bp homologous arms flanking a non-addressed target sequence were co-transfected with the reference system (Fig. 5A). After 24 h of incubation, fluorescence microscopy was performed to visualize PARP1 mediated changes in GFP2 fluorescence of the BRET reporter system, indicative of reading frame disruption and restoration (Fig. 5B). Cells transfected with the BRET reporter *CLN3* reference +gRNA/+Cas9 complex exhibited a clear reduction in GFP2 fluorescence compared to control cells transfected with Cas9 alone and without the guiding RNA. As expected from the previous results on hyperactive NHEJ, PARP1 KO 2 and 4 led to a further decrease in fluorescent positive cells, while the addition of the HDR repair template restored the signal. The PARP1 downregulation in presence of the HDR repair template showed no visible in-or decrease in GFP positive cells, while PARP1 overexpression suggested a decreased amount of positive cells (Fig. 5B). To quantify these effects a BRET assay was performed again to assess changes in BRET ration upon HDR template, PARP1 gRNA/Cas9 knockout or overexpression plasmid addition. The differences in the resulting frameshift rate with and without the HDR repair template is represented as the HDR activity and the PARP1 mediated change in the HDR rate change [%] (Fig. 5C).

PARP1 overexpression resulted in a reduction in GFP2 fluorescence of the BRET reporter system and thus in the HDR activity experiment, suggesting a negative regulatory role of PARP1 in HDR-mediated repair. None of the PARP1 knockouts could significantly alter the HDR integration efficiency, whereas PARP1 overexpression led to a highly significant reduction in HDR efficiency by 10.3 ± 4.8 % (Fig. 5C). So we could show a highly significant (***p < 0.001) increase of HDR meditated repair template integration into the targeted locus at the BRET reporter reference upon PARP1 overexpression.

## 4 Discussion

Following a DNA-DSB induced by endonucleases such as Cas9, different repair pathways compete for access to the free DNA ends. Pathway choice is influenced by the cell cycle stage, the local chromatin environment, and the sequence context at the target site. [17], [18], [19] In proliferating cells, repair options span the entire cell cycle, whereas non-proliferating cells such as retinal photoreceptors remain permanently arrested in the G0 phase. As a result, HDR-mediated integration is considered largely inefficient in these cells due to low expression of homologous recombination factors. In mice, HDR-associated proteins are downregulated in photoreceptors shortly after birth. However, studies demonstrate that HDR activity persists in retinal organoids under *in-vitro* culture and in neuronal progenitors during differentiation. [20] This suggests that redirecting repair pathway choice from error-prone NHEJ toward HDR or MMEJ, both of which can support precise template-driven integration via micro- or macro-homologies, may be feasible through DNA repair pathway engineering. To test this, and given the involvement of PARP1 in all three major DSB repair mechanisms, we analyzed the effects of PARP1 downregulation and overexpression using luminescent and fluorescent reporter assays in HEK293T cells.

### 4.1 Impact of PARP1 modulation on NHEJ

Our data provide strong evidence that CRISPR/Cas9-mediated downregulation of PARP1 significantly increases frameshift events, consistent with an elevated frequency of NHEJ-induced indels. In contrast, transient overexpression of PARP1 suppressed NHEJ activity and reduced mutagenic outcomes in our assay. Mechanistically, PARP1 and the Ku complex are known to compete for binding at free DNA ends; thus, PARP1 depletion likely facilitates greater Ku occupancy at DSB sites, thereby enhancing NHEJ. An alternative explanation is that the absence of PARP1 alters the recruitment dynamics of core NHEJ factors. These observations are in line with previous reports in PARP1 knockout hematopoietic stem and progenitor cells (HSPCs) as well as in immortalized retinal pigment epithelial (RPE) cells, where hyperactive NHEJ was observed and subsequently normalized upon PARP1 re-expression. [21]

### 4.2 PARP1and MMEJ integration efficiency

The role of PARP1 in MMEJ remains controversial. While some studies suggest that PARP1 is dispensable, others propose that it actively initiates MMEJ via the recruitment of XRCC1 and Ligase III. [22] Our data indicate that PARP1 overexpression does not affect MMEJ-mediated repair template integration. However, PARP1 downregulation significantly increased template integration**,** as reflected by elevated luciferase activity following substrate addition**.** Interestingly, even partial PARP1 depletion enhanced MMEJ efficiency, and a complete knockout further increased targeted integration.

### 4.3 PARP1 and HDR regulation

We further examined the effect of PARP1 levels on HDR activity. PARP1 interacts with the MRN complex (MRE11-RAD50-NBS1), facilitating 3′ overhang generation, which is essential for HDR initiation. [17] Our findings confirm that PARP1 downregulation had no measurable effect on HDR efficiency. A plausible explanation is that PARP2, a functional homolog of PARP1, compensates for its loss, thereby maintaining HDR function. [3] Notably**,** PARP1 overexpression significantly reduced HDR activity in our BRET assay, consistent with previous reports showing that excessive PARP1 accumulation inhibits sister chromatid exchange, thereby negatively affecting HDR efficiency. [1]

### 4.4 Implications for DNA repair pathway engineering in genome editing

Although CRISPR/Cas9 would not be used to permanently downregulate or knock out PARP1 in living organisms, these modifications provide valuable insights into DNA repair mechanisms *in-vitro*. In a therapeutic context, DNA repair pathway engineering should rely on transient modulation, for which siRNA, small-molecule inhibitors, or neutralizing antibodies are preferable approaches. [23], [24], [25] Based on our findings, an optimal strategy to suppress mutagenic NHEJ while preserving MMEJ and HDR activity would involve PARP1 overexpression**.** Transient overexpression of PARP1 effectively reduced NHEJ without impairing MMEJ. However**,** PARP1 depletion, despite enhancing MMEJ, also promoted NHEJ, making it unsuitable for therapeutic genome editing**.** This is particularly relevant for non-proliferating photoreceptor cells, where HDR is highly inefficient. In such contexts, a controlled transient expression of PARP1 could suppress NHEJ-driven mutagenesis while preserving MMEJ activity**,** thereby minimizing unintended genetic modifications.

## 5 Conclusion

Our study provides new insights into the role of PARP1 in regulating DNA-DSB repair pathway choice in proliferating cells and its implications for genome editing. Through systematic modulation of PARP1 expression, we found that transient overexpression suppresses NHEJ, thereby reducing mutagenic indel formation, without impairing MMEJ or HDR. In contrast, PARP1 depletion increased both NHEJ and MMEJ activity, suggesting that although MMEJ can function as an alternative repair route, the parallel rise in error-prone NHEJ limits the therapeutic utility of PARP1 downregulation. Taken together, these results highlight PARP1 as a tunable regulator of DSB repair and point to its potential for enhancing the precision and safety of therapeutic genome editing.

## CRediT authorship contribution statement

**Judith Schneider:** Methodology, Investigation. **Türk Franziska:** Investigation. **Lyubomyr Lytvynchuk:** Writing – review & editing. **Knut Stieger:** Writing – review & editing, Supervision, Project administration, Funding acquisition, Conceptualization. **Tobias Wimmer:** Writing – original draft, Supervision, Methodology, Investigation, Formal analysis, Conceptualization. **Fatimah Alabudeeb:** Writing – review & editing, Investigation.

## Declaration of Generative AI and AI-assisted technologies in the writing process

During the preparation of this work the author(s) used ChatGPT in order to improve grammar and readability. After using this tool/service, the author(s) reviewed and edited the content as needed and take(s) full responsibility for the content of the publication.

## Declaration of Competing Interest

None of the authors has a financial interest to declare.T.W. and K.S. are holding a patent of the BRET reporter system.
