# Supplementary material for: Influence of PARP1 on CRISPR/Cas9 induced double strand break repair in proliferating cells
Source: Comput Struct Biotechnol J. 2025 Oct 2;27:4282–9. doi: 10.1016/j.csbj.2025.10.001 (PMC12538023; doi:10.1016/j.csbj.2025.10.001)
Supplement: Supplementary file 1 — Supplementary material [file mmc1.docx]

Supplements:

Supp.: Cas9 target and gRNA sequences, micro-homologous sequences and PCR primer sequences used in this study.

Figure 1:


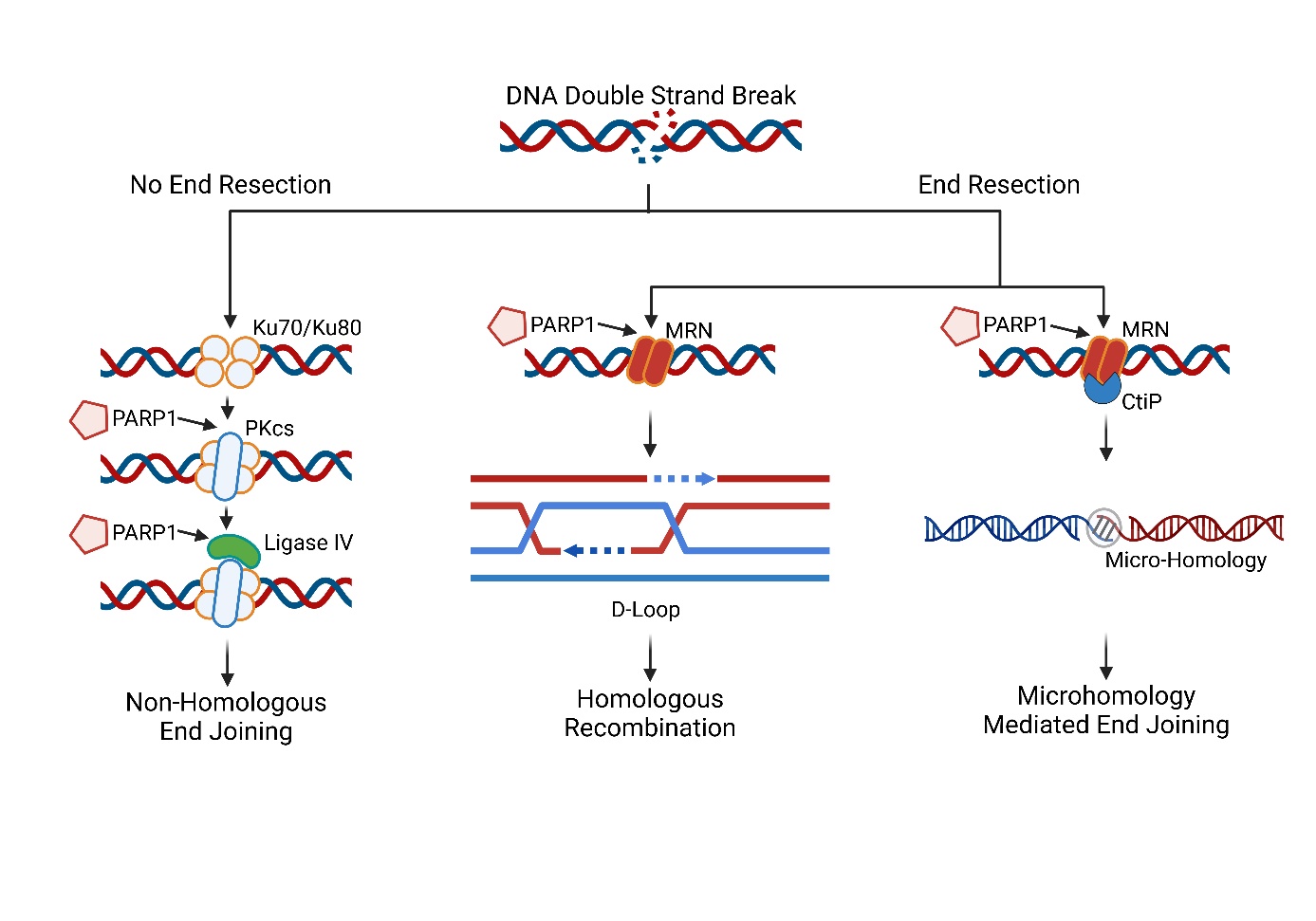


Supplements:

|  | Sequence (5‘-3‘)PAM |
| --- | --- |
| Target DNA 1 | GTTACCCACTCCTTCCGGTT GGG |
| gRNA 1 | GTTACCCACTCCTTCCGGTT |
| Target DNA 2 | GCCACGGAGGCGCTGGTTTC TGG |
| gRNA 2 | GCCACGGAGGCGCTGGTTTC |
| Target DNA 3 | CTGACCCGAGCATTCCTCC AGG |
| gRNA 3 | CTGACCCGAGCATTCCTCC |
| Target DNA 4 | AGAAACCAGCGCCTCCGTGG CGG |
| gRNA 4 | AGAAACCAGCGCCTCCGTGG |
| Target DNA 5 | CACGGAGGCGCTGGTTTCTG GGG |
| gRNA 5 | CACGGAGGCGCTGGTTTCTG |

|  | Sequence (5‘-3‘) PAM |
| --- | --- |
| BRET *CLN3* In8T3 (Target DNA) | TGTGGGGCTTGCTCACCTCC AGG |
| Px459 *CLN3* In8T3 (gRNA) | TGTGGGGCTTGCTCACCTCC |

|  | | Sequence (5‘-3‘) |
| --- | --- | --- |
| ORF15-gRNA1 | | GTCAGGGATACCAGAGGAGC |
| ORF15-gRNA2 | | TCCAGAATCGTTCGGAGCCT |
| MH-Luc-gRNA1 | | GAGCGCCACCATGGTGAGCA |
| MH-Luc-gRNA2 | | GGCCACAAGTTCAGCGTGTC |
| MH-10 bp | MH1 | TTCGATCTTA |
|  | MH2 | GACTGCTGAC |
| MH-15 bp | MH1 | CAAAGTTCGATCTTA |
|  | MH2 | GACTGCTGACATGAC |
| MH-30 bp | MH1 | CAACCAGATTTTTCC CAAAGTTCGATCTTA |
|  | MH2 | GACTGCTGACATGACGTCGAATGCGTAGCA |

|  | Sequenz (5‘-3‘) PAM |
| --- | --- |
| In6T4 HDR Template (Target DNA) | CAGGCCCTTGTTCGGACTGC TGG |
| HDR PCR Primer I | ATT GTC CGC AAC TAC AAC GCC TAC |
| HDR PCR Primer II | TCG GGG CGG ATG TAC ACG TTG |

**Declaration of generative AI and AI-assisted technologies in the writing process**

During the preparation of this work the author(s) used ChatGPT in order to improve grammar and readability. After using this tool/service, the author(s) reviewed and edited the content as needed and take(s) full responsibility for the content of the publication.
